# Dataset of coal bio-gasification and coalbed methane stimulation by single well nutrition injection in Qinshui anthracite coalbed methane wells

**DOI:** 10.1016/j.dib.2022.108353

**Published:** 2022-06-04

**Authors:** Dong Xiao, Mohamed Keita, Cong Zhang, Enyuan Wang, Norberto Daniel Diaz, Junyong Wu, Hailun He, Jing Ma, Essono Oyono Julien

**Affiliations:** a CUMT-UCASAL Joint Research Center for Biomining and Soil Ecological Restoration, State Key Laboratory of Coal Resources and Safe Mining, China University of Mining and Technology, Xuzhou, Jiangsu 221116 China; b Shanxi Coal Bed Methane Company of PetroChina, Jincheng, Shanxi 048000, China; c Key Laboratory of Gas and Fire Control for Coal Mines, China University of Mining and Technology, Xuzhou, Jiangsu 221116 China; d CUMT-UCASAL Joint Research Center for Biomining and Soil Ecological Restoration, Universidad Católica de Salta, Salta A4400EDD Argentina; e School of Life Science, Central South University, Changsha, Hunan 410083 China; f School of Environment Science and Spatial Informatics, China University of Mining and Technology, Xuzhou, Jiangsu 221116 China

**Keywords:** Anthracite, Coal gasification, Bio-methane, *Methanogen* spp., Coal-bed methane well, *In-situ* pilot test

## Abstract

In-situ coal bio-gasification can be defined as one of the coal bio-mining methodology that fully utilizes the methanogenic bacteria in coal to review the current findings, namely anaerobic digestion of organic components. The following experiment has been done in regards, one vertical well and one multi-branch horizontal well were used as experiment wells and two vertical wells were used as control wells, the pilot test was carried out with single well nutrition injection method. By applying the above mentioned method, the concentration of Cl^−^ ion and number altered in *Methanogen* spp. were used to trace nutrition diffusion. Furthermore, technical implementation results analysis has been made with the observation of CH_4_ production changes and coal bed biome evolution.

Gas production rates in each well were monitored by using the FLLQ gas roots flow mete. The concentration of CH_4_ and CO_2_ were evaluated by using the Agilent 7890A gas chromatograph, on the other hand, concentrations of Cl^−^ were determined by the application of ICS-1100 ion chromatography system. The F_420_ fluorescence method was adopted to test for the presence of methanogenic bacteria. In the interim of the completion stage, the study stated that the bacterial diversity of underground water of Z-7H well has a high pass sequence with the experimental period of 814 days. Gas production data in Z-159 and Z-7H wells showed the gasification of coal lasted 635 and 799 days, yielded 74817 m^3^ and 251754 m^3^ coalbed methane, respectively. Furthermore, experimental data presented that one time nutrition injection in anthracite coalbed methane wells achieved an average of 717 days of continuous gas production among all experimental wells. The above fore-said study dedicated the significance of native bacterial fermentation, as it proven the fact that anthracite can be applied to accomplish coal bio-gasification and coalbed methane production stimulation in-situ.

## Specifications Table

**Table:** 

Subject	Bioenergy, Mining Engineering
Specific subject area	Coal bio-mining and coalbed methane production stimulation.
Type of data	• Table • Graph • Figure • Biodiversity test data
How the data were acquired	Gas production rates in each well were monitored using FLLQ gas roots flow mete (model: FLLQ, Fuma, China). The concentration of CH_4_ and CO_2_ were analyzed using Agilent 7890A (Agilent, America) gas chromatograph. Concentrations of Cl^−^ were identified using an ICS-1100 ion chromatography system (Thermo Scientific Dionex, Bannockburn, America). The number of methanogens was counted by fluorescence microscope (model: BX41, Olympus, Tokyo, Japan), and the excitation light wavelength was 420 nm (F_420_ fluorescence). Total genomic DNA was extracted from 1 mL concentrated underground water samples using E.A.N.A. Soil DNA Kit (OMEGA, Georgia, GA, USA). The V4 region of 16S rRNA gene was amplified with polymerase chain reaction (PCR) using primers 515F (5’- GTG CCA GCM GCC GCG GTAA - 3’) and 806R (5’- GGA CTA CHV GGG TWT CTA AT - 3’). 16S rRNA gene libraries were sequenced using an Illumina MiSeq (San Diego, CA, USA) platform and the sequencing data were base-called and demultiplexed using MiSeq Reporter v.1.8.1 (Illumina, SanDiego, CA, USA) with default parameters.
	The adapter sequences and low quality reads were trimmed away from the raw reads with Trimmomatic v.0.32. Venn diagram analysis software: R language (version 3.3.1) tools for statistics and graphing. Evolutionary tree software: IQ-TREE (version 1.6.8 http://www.iqtree.org/). Circos charting software: Circos-0.67-7 (http://circos.ca/). Heatmap software: R language (version 3.3.1) vegan package. Sample difference analysis: stats package for R (version 3.3.1) and scipy package for python Figures were drawn using Origin (OriginPro 2018C).
Data format	Raw Analyzed
Description of data collection	The data of daily gas production for each experimental well were monitored, and the data collection was processed for 814 days. The concentration differences of CH_4_ and CO_2_ in coalbed gas before and after experiments were analyzed. The differences in Cl^−^ ion concentration and methanogenic bacteria number among the experimental wells and the surrounding control wells before and after the experiment were analyzed.
Data source location	● City/Town/Region: Jincheng, Shanxi province ● Country: China ● This experiment was carried out in a multi-branch horizontal well and a vertical well. The horizontal well identified as Z-7H (GPS coordinates: 35.716, 106.475) and Z-159(GPS coordinates: 35.710, 112.472). Z-163, Z-167wells were control wells which were located beside the test wells.
Data accessibility	**Basic Data**: Repository name: Mendeley Data Data identification number: 10.17632/pj5jk82w55.6 Direct URL to data: https://data.mendeley.com/datasets/pj5jk82w55/draft?a=10550b6a-bc56-47e6-aa51-aaec9590c611 **Biodiversity Data** Repository name: NCBI Sequence Read Archive Accession number: PRJNA828322
Related research article	Xiao D, Zhang C, Wu J, et al. Primary studies on the effect of coal bio-gasification in situ in the Qinshui basin[J]. Journal of Petroleum Exploration and Production Technology, 2021: 1-10. 10.1007/s13202-021-01396-8

## Value of the Data

• From 2015 to 2017, the first successful pilot test of in-situ bio-gasification of coal was carried out in anthracite coal seams in China. This experiment processed a total of 814 days.
• The daily gas production data contains one multi-branch horizontal well and one vertical well along the whole process. It provides valuable data for the research and process design of microbial gasification of coal, especially for high-rank coal.
• The data are relevant for researchers seeking for a classification system to coal bio-gasification and coalbed methane stimulation.
• The data can be used for further experiments such as microbial enhancement coalbed methane, H_2_ production from coal bio-fermentation, microbial dredging of coal micro pores, which can correlate coal vs. bio-fermentation.

## 1 Data Description

Datasets in this paper were used in the research article “Primary Studies on the Effect of Coal Bio-gasification In-situ in the Qinshui Basin [1]”. The datasets consist of the experimental wells locations, 635 days and 799 days of daily gas production data of vertical and horizontal experiment well correspondingly. The datasets recorded modifications in critical characteristic indexes of coal microbial degradation. Indicators comprises: coalbed gas concentration, Cl^−^ ion concentration, methanogenic bacteria number changes in underground water and biodiversity (Table 1).

**Table 1:** Data file description.

Data file	Description
Table 2	CH_4_, CO_2_ and N_2_ concentration changes in experimental wells and control wells before and after the experiment.
Table 3	The methanogenic number changes before and after the experiment.
Table 4	The molar Cl^−^ concentration in experimental wells and control wells before and after the experiment.
Fig. 1	Experiment location.
Fig. 2	Photographs of media injection in site.
Fig. 3	Fig. 3 A to E plot chart of daily gas production data of experimental wells and control wells in experiment.
Fig. 4	Fluorescence characteristics of coal seam methanogenic bacteria after cultivation
Fig. 5	Fig. 5 A to F biodiversity difference analysis of unground water before and after the experiment.
Data 1	Daily gas production raw data and relative gas production data of experimental wells and control wells.

## 2 Experimental Design, Materials and Methods

### 2.1 Medium Preparation

150 m^3^ methanogenic bacteria culture medium were prepared for both Z-7H well and Z-159 well experiment. The concentrations of the medium compounds (kg/m^3^) were: yeast extract, 0.50; NaHCO_3_, 0.05; NH_4_Cl, 2.30; KH_2_PO_4_, 1.30; K_2_HPO_4_, 0.70; NaCl, 0.05; MgSO_4_•7H_2_O, 0.20; CaCl_2_•2H_2_O, 0.05 [2].

The final pH of medium was 6.80, and the Cl^−^ ion concentration was 44.59 mmol/L.

### 2.2 Nutrition Injection

**Fig. 1:**
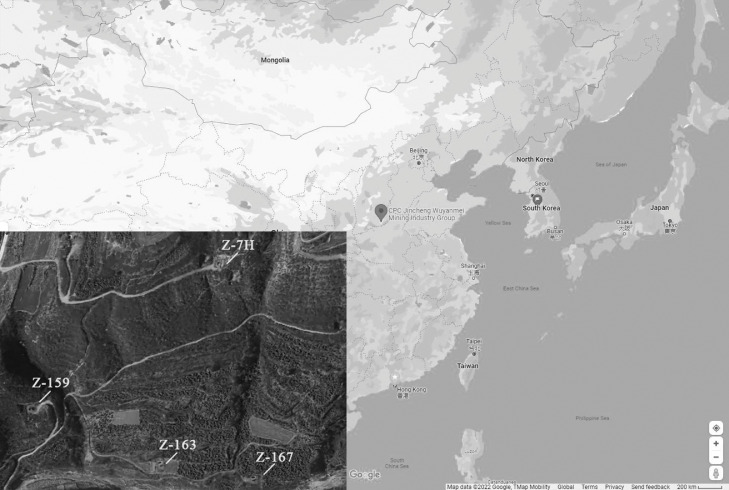
Experiment location in China. *Digital map via Google Earth.*

This experiment was carried out in a multi-branch horizontal well (Z-7H, GPS coordinates: 35.716, 106.475) along with a vertical well (Z-159, GPS coordinates: 35.710, 112.472). Simultaneously, Z-163, Z-167, which were selected as control wells are currently located beside the test wells (Fig. 1).

**Fig. 2:**
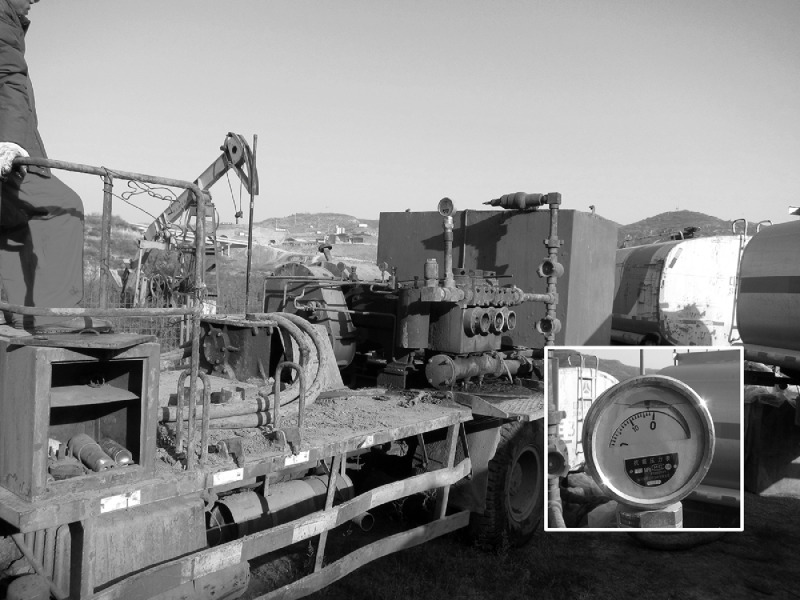
On-site media injection and pressure control.

In accordance to the type of experimental wells, 100 m³ medium was prepared for Z-7H well, and 30 m³ medium was prepared for Z-159 well. The medium injection was performed with a fracturing truck (4150 8 × 8, Benz, Germany), the pressure was adjusted and maintained at less than 4.00 MPa (Fig. 2). The Z-159 well and Z-7H well were sealed after nutrition injected on March 11 and 26, 2015.

### 2.3 CBM wells Gas Productivity Data Collection and Relative CBM Yield Calculation Method

**Fig. 3:**
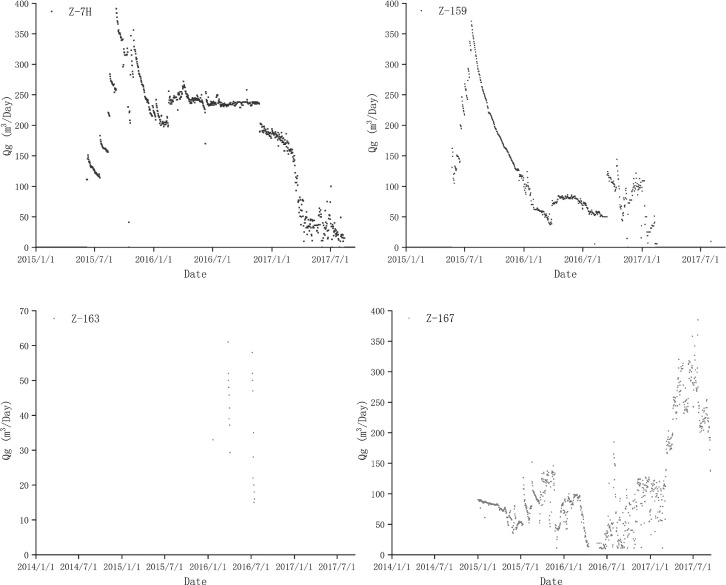
The daily gas production plot chart of the experimental wells and the control wells. Fig. 3 A and B are experimental wells, marked with blue notes. Fig. 3 C and D were control wells, marked with read notes.

CBM production rates in each well were monitored on line using a gas roots flow meter (model: FLLQ, Fuma, China). Measurement accuracy of the FLLQ gas roots flow meter was class 1.0, along with the low range of this meter was 0.6–400 m³/h. The sensor counted the total gas production every 24 h and automated the data to the host computer on a regular basis. The cumulative gas production accounted as the daily gas production (Qg, m^3^/Day) of experimental wells (Fig. 3, data 1).

### 2.4 CBM Wells Gas Composition Data Collection

The concentration of CH_4_, CO_2_, and N_2_ were analysed prior to and following to the study using Agilent 7890A gas chromatograph (Agilent, America). Subsequent indexes have been fixed in accordance to the experiment, the nitrogen (carrier gas) flow rate was fixed at 1 mL/min, injection port was maintained at 150 °C, oven temperature was 25 °C and the TCD was operated at 200 °C. Retention time for methane was 3.76 min and 5.0 min for CO_2_. Calibration standards consisting of 40% methane, 20% CO_2_, 10% hydrogen and 30% nitrogen were injected at atmospheric pressure to contribute the calibration plot (Table 2).

**Table 2:** Gas component concentration table.

	CH_4_ Concentrations % Vol					
	Before			After		
	data	Max	min	data	max	min
Z-7H	97.76%			79.92%		
	98.01%			80.06%		
	97.70%			79.86%		
Average	**97.82%**	**0.19%**	**0.12%**	**79.95%**	**0.11%**	**0.09%**

Z-159	97.17%			86.11%		
	97.25%			88.06%		
	97.20%			86.82%		
Average	**97.21%**	**0.04%**	**0.04%**	**87.00%**	**1.06%**	**0.89%**

Z-163	98.13%			98.12%		
	97.98%			96.71%		
	97.94%			97.52%		
Average	**98.02%**	**0.11%**	**0.08%**	**97.45%**	**0.67%**	**0.74%**

Z-167	97.50%			98.03%		
	97.62%			97.55%		
	97.43%			97.88%		
Average	**97.52%**	**0.10%**	**0.09%**	**97.82%**	**0.21%**	**0.27%**

	CO_2_ Concentrations % Vol					
Z-7H	2.09%			19.96%		
	1.97%			19.83%		
	2.27%			20.12%		
**Average**	**2.11%**	**0.16%**	**0.14%**	**19.97%**	**0.15%**	**0.14%**

Z-159	2.63%			13.71%		
	2.66%			11.85%		
	2.66%			13.08%		
**Average**	**2.65%**	**0.01%**	**0.02%**	**12.88%**	**0.83%**	**1.03%**

Z-163	1.83%			1.80%		
	2.02%			3.06%		
	2.00%			2.46%		
**Average**	**1.95%**	**0.07%**	**0.12%**	**2.44%**	**0.62%**	**0.64%**

Z-167	2.41%			1.84%		
	2.30%			2.40%		
	2.37%			1.85%		
**Average**	**2.36%**	**0.05%**	**0.06%**	**2.03%**	**0.37%**	**0.19%**

	N_2_ Concentrations % Vol					
Z-7H	0.15%			0.12%		
	0.02%			0.11%		
	0.03%			0.02%		
Average	**0.07%**	**0.08%**	**0.05%**	**0.08%**	**0.04%**	**0.06%**

Z-159	0.20%			0.18%		
	0.09%			0.09%		
	0.14%			0.10%		
Average	**0.14%**	**0.06%**	**0.05%**	**0.12%**	**0.06%**	**0.03%**

Z-163	0.04%			0.08%		
	0.00%			0.23%		
	0.06%			0.02%		
Average	**0.03%**	**0.03%**	**0.03%**	**0.11%**	**0.12%**	**0.09%**

Z-167	0.09%			0.13%		
	0.08%			0.05%		
	0.20%			0.27%		
Average	**0.12%**	**0.08%**	**0.04%**	**0.15%**	**0.12%**	**0.10%**

Since the carrier gas is N_2_, hence the N_2_ concentration in the test can only be used as a referencing value exclusively to indicate the possibility of air mixing into the gas sample.

### 2.5 Methanogens Counts

**Fig. 4:**
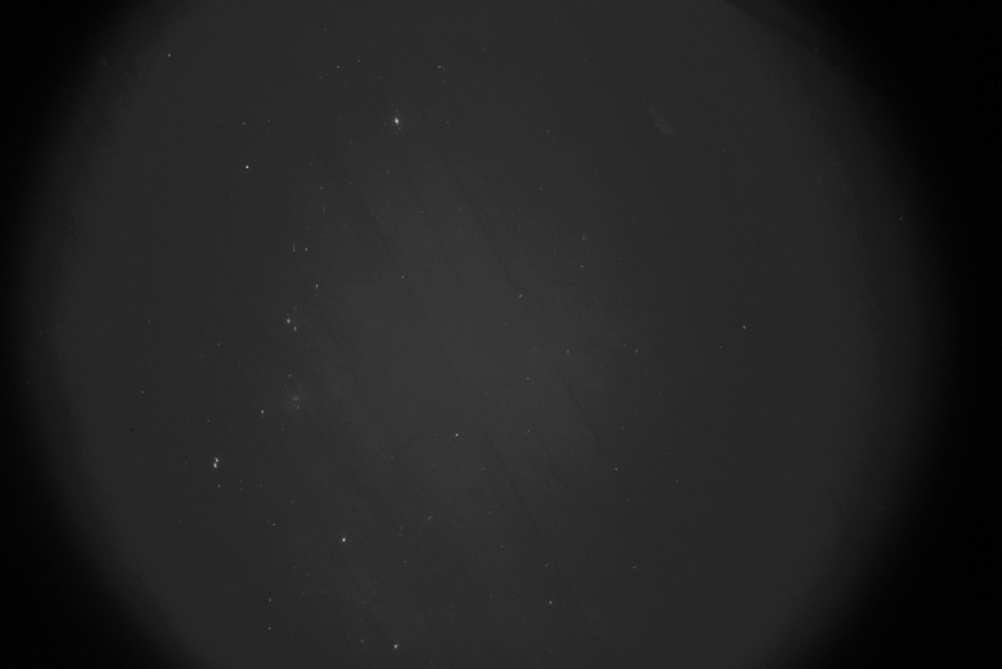
Fluorescence characteristics of methanogens F_420_.

Methanogens counting were performed using an Olympus BX41 (Olympus, Japan) fluorescence microscope at 40 × with a blood cell counting plate. The fluorescence excitation centre wavelength is F_420_, and the filter block wavelength is F_460_-_480_ [3] (Fig. 4, Table 3)

**Table 3:** Methanogenic number changes before and after experiment.

	Methanogen Number × 10^5^ per mL					
	Before			After		
	data	max	min	Data	max	min
Z-7H	1.00			740.00		
	1.00			945.00		
	0.50			595.00		
Average	**0.83**	**0.17**	**0.33**	**760.00**	**185.00**	**165.00**

Z-159	1.00			485.00		
	0.50			690.00		
	0.50			505.00		
Average	**0.67**	**0.33**	**0.17**	**560.00**	**130.00**	**75.00**

Z-163	0.50			0.00		
	0.50			1.00		
	1.50			0.00		
Average	**0.83**	**0.67**	**0.33**	**0.33**	**0.67**	**0.33**

Z-167	1.00			0.50		
	1.00			2.00		
	1.50			1.00		
Average	**1.17**	**0.33**	**0.17**	**1.17**	**0.83**	**0.67**

**Table 4:** Cl^−^ concentration before and after the experiments.

	Cl^–^ Concentration mmol/L					
	Before			After		
	data	max	min	data	max	min
Z-7H	2.03			3.67		
	1.88			4.23		
	2.17			4.14		
Average	**2.03**	**0.14**	**0.15**	**4.01**	**0.22**	**0.34**

Z-159	1.93			5.86		
	2.01			4.98		
	1.81			5.38		
Average	**1.92**	**0.09**	**0.11**	**5.41**	**0.45**	**0.43**

Z-163	1.99			1.97		
	2.11			1.81		
	1.84			2.26		
Average	**1.98**	**0.13**	**0.14**	**2.01**	**0.25**	**0.20**

Z-167	1.86			1.93		
	1.97			2.07		
	2.06			2.13		
Average	**1.96**	**0.10**	**0.10**	**2.04**	**0.09**	**0.11**

### 2.6 Cl^−^ Ion Concentration Data Collection

Concentrations of Cl^−^ were identified prior and following to the experiment using an ICS-1100 ion chromatography system (Thermo Scientific Dionex, Bannockburn, America). The underground water sample was collected in wells Z-159, Z-163, Z-167, and Z-7H with the acquisition of aseptic, anaerobic 50 mL tubes. In addition to high-speed multifunction centrifugation (J2-MC, Beckman Instruments, Fullerton, America) and 0.22 μm filter membrane were employed to separate the suspended particles and microbes in underground water samples.

### 2.7 Ground Water Collection, DNA Extraction, PCR, and Sequencing Method

(1) 100 mL of underground water in the Z-7H well was collected in March 20, 2015 (Prior to nutrition injection) and in March 25, 2017 (CBM yield decreases following to the completion of the experiment). Bacteria was concentrated to 1 mL by centrifugation (J2-MC, Beckman Instruments, America) and temporary stored in cryovials at -80 °C (refrigerator type: DW-86L728J, Haier, China).
(2) Total genomic DNA was extracted from 1 mL concentrated underground water samples using E.A.N.A. Soil DNA Kit (OMEGA, Georgia, USA) following the manufacturer's instructions.
(3) The V4 region of 16S rRNA gene was amplified with polymerase chain reaction (PCR) using primers 515F (5’- GTG CCA GCM GCC GCG GTAA - 3’) and 806R (5’- GGA CTA CHV GGG TWT CTA AT - 3’) [4,5].
(4) Each 20 μL PCR reaction composed of 2 ng of template DNA, 0.2 μM primers, 0.2 mM dNTP, 2 μL 10 × Pfu Buffer with MgSO_4_ (Applied Thermo, USA), with H_2_O up to 20 μL. The DNA amplification was performed under the following cycling conditions: 1 cycle of 2 min at 95 °C, followed by 30 cycles with 30 s at 95 °C, 30 s at 55 °C and 1 min at 72 °C, following a final extension period of 5 min at 72 °C.
(5) Prior to the sequencing on the Illumina Miseq sequencing platform, the V4 region was amplified by adding sample-specific 10-base barcodes and universal sequencing tags by Sample-Specific PCR protocol. The PCR procedure was as follows: 1 cycle of 95 °C at 2 min, 15 cycles of 95 °C at 15 s, 60 °C at 30 s, 68 °C at 1 min, the final stage was 1 cycle of 68 °C at 3 min.
(6) Equal volume of each barcoded product was pooled into amplicon libraries and purified using Agencourt AMPure XP system (Beckman Coulter, USA) thenceforth, examined on Agilent Bioanalyzer 2100 for product size distribution. The purified libraries were quantified with Qubit® dsDNA HS Assay Kit (Life Technologies, USA) and used for sequencing.
(7) High-throughput sequencing was performed by BGI, the adapter sequences and low quality reads were trimmed away from the raw reads with Trimmomatic v.0.32 [6].

### 2.9 Underground Water Biodiversity Data Analysis

The sequencing data was imported into the Meggie Gene Cloud Platform for subsequent analysis.

Venn diagram analysis selects OTU samples with a similar level of 97%, statistics and graphing software: R language (version 3.3.1) (Fig. 5 A).

**Fig. 5:**
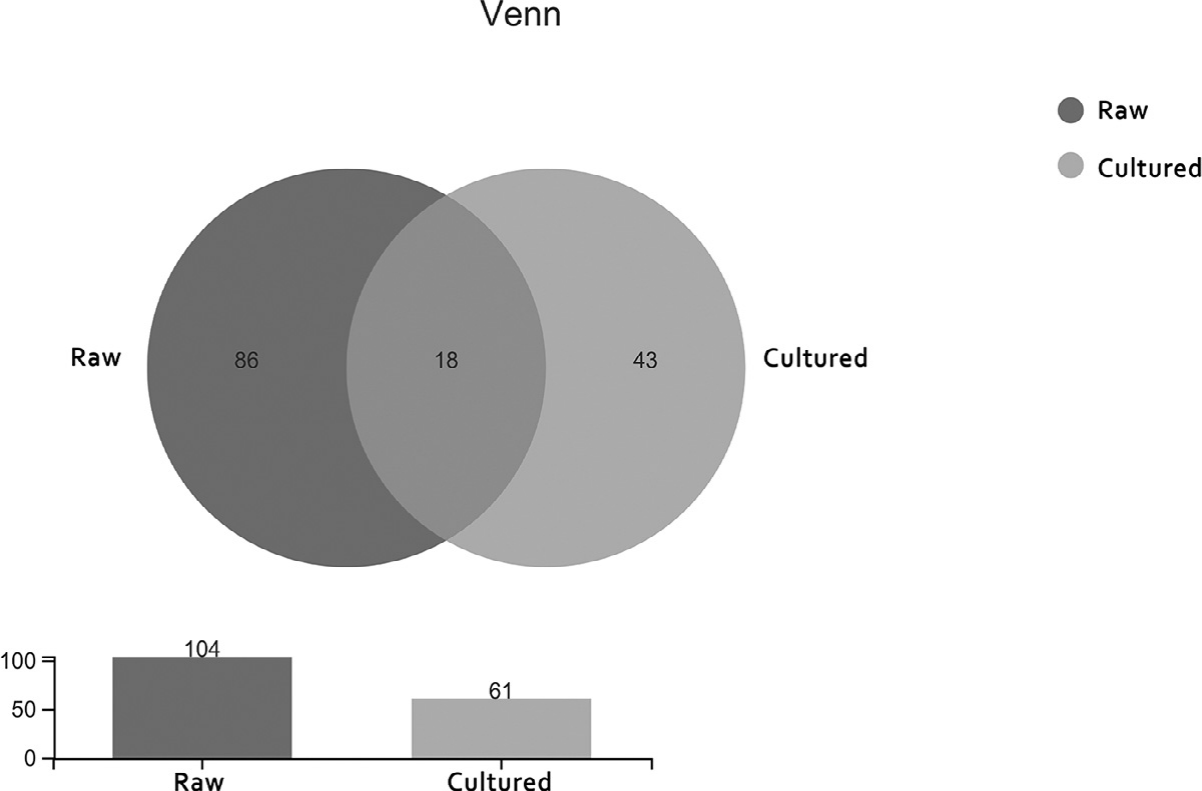

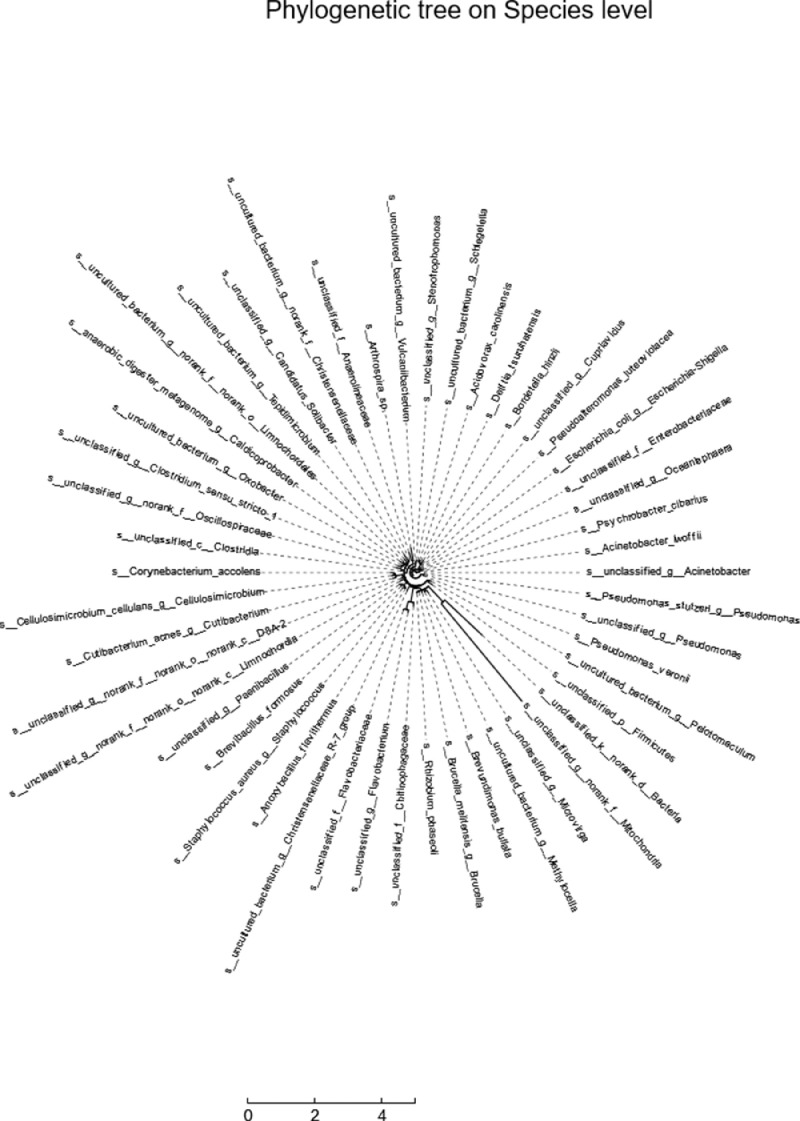

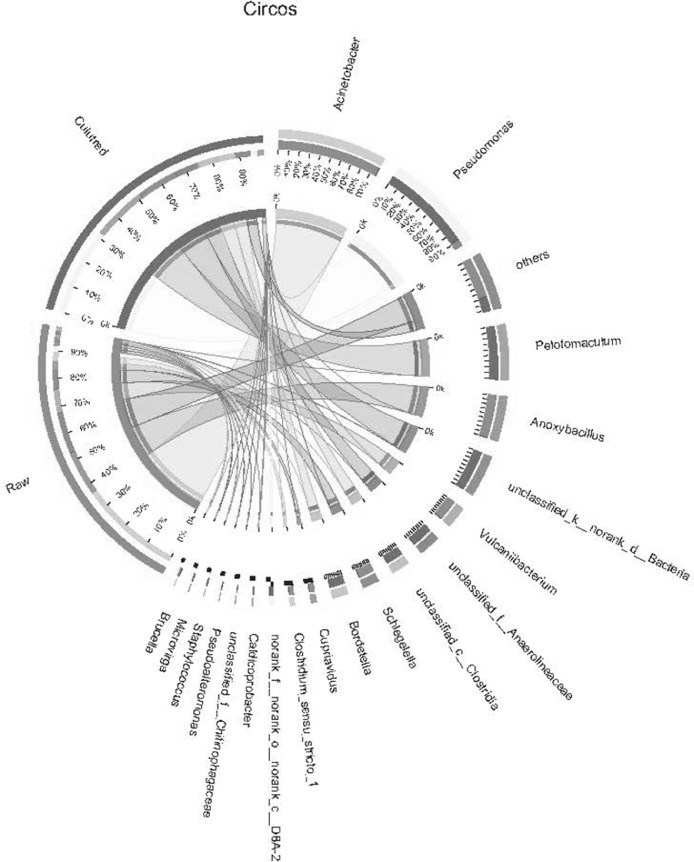
Two-samples biodiversity analysis.

**Fig. 5:**
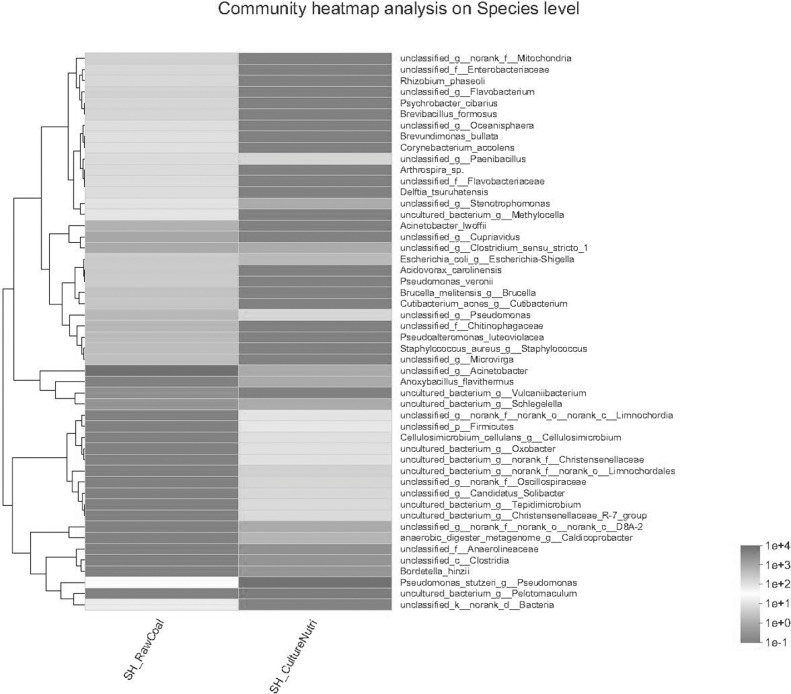
Continued.

**Fig. 5:**
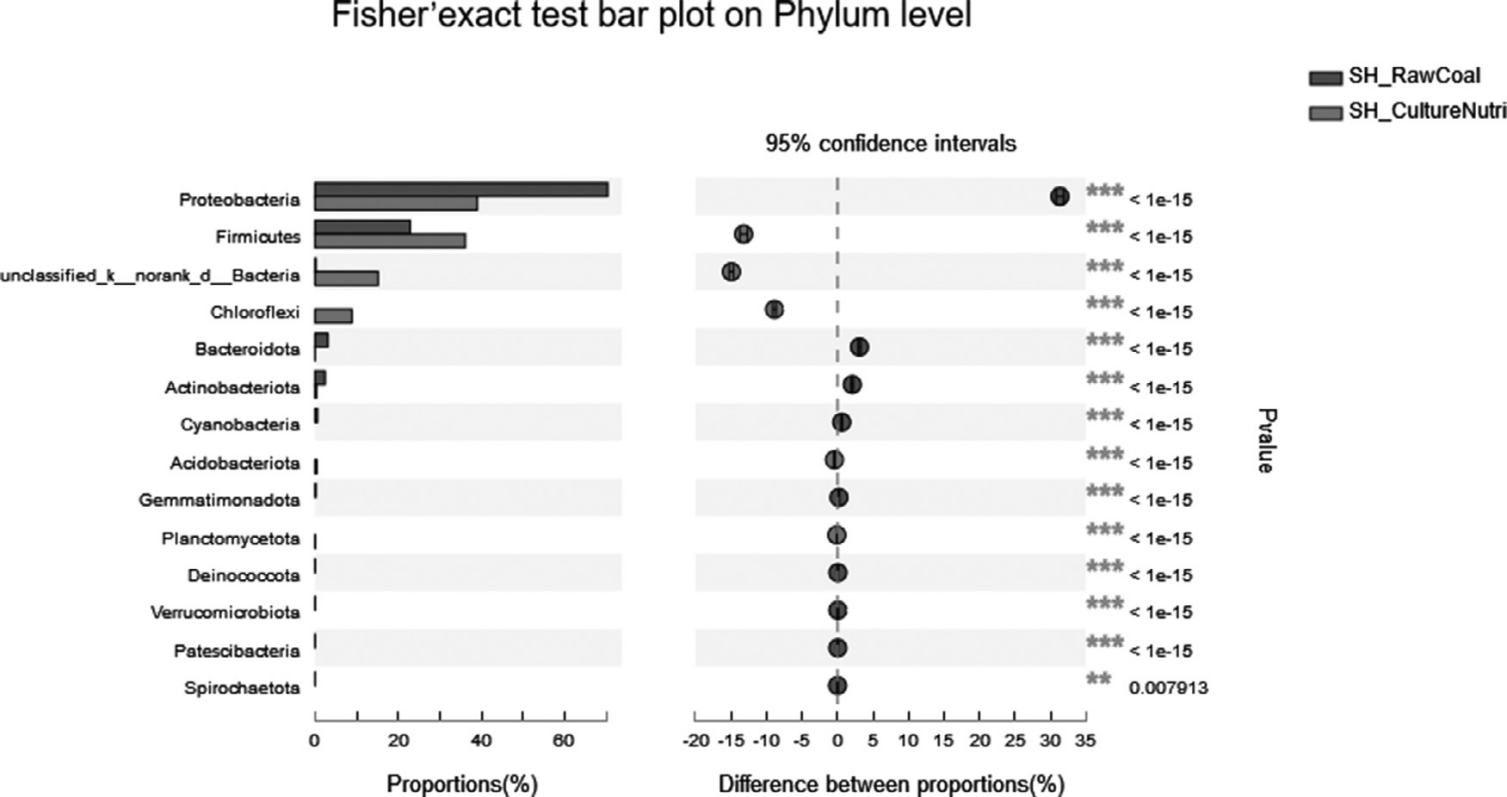
Continued.

**Fig. 5:**
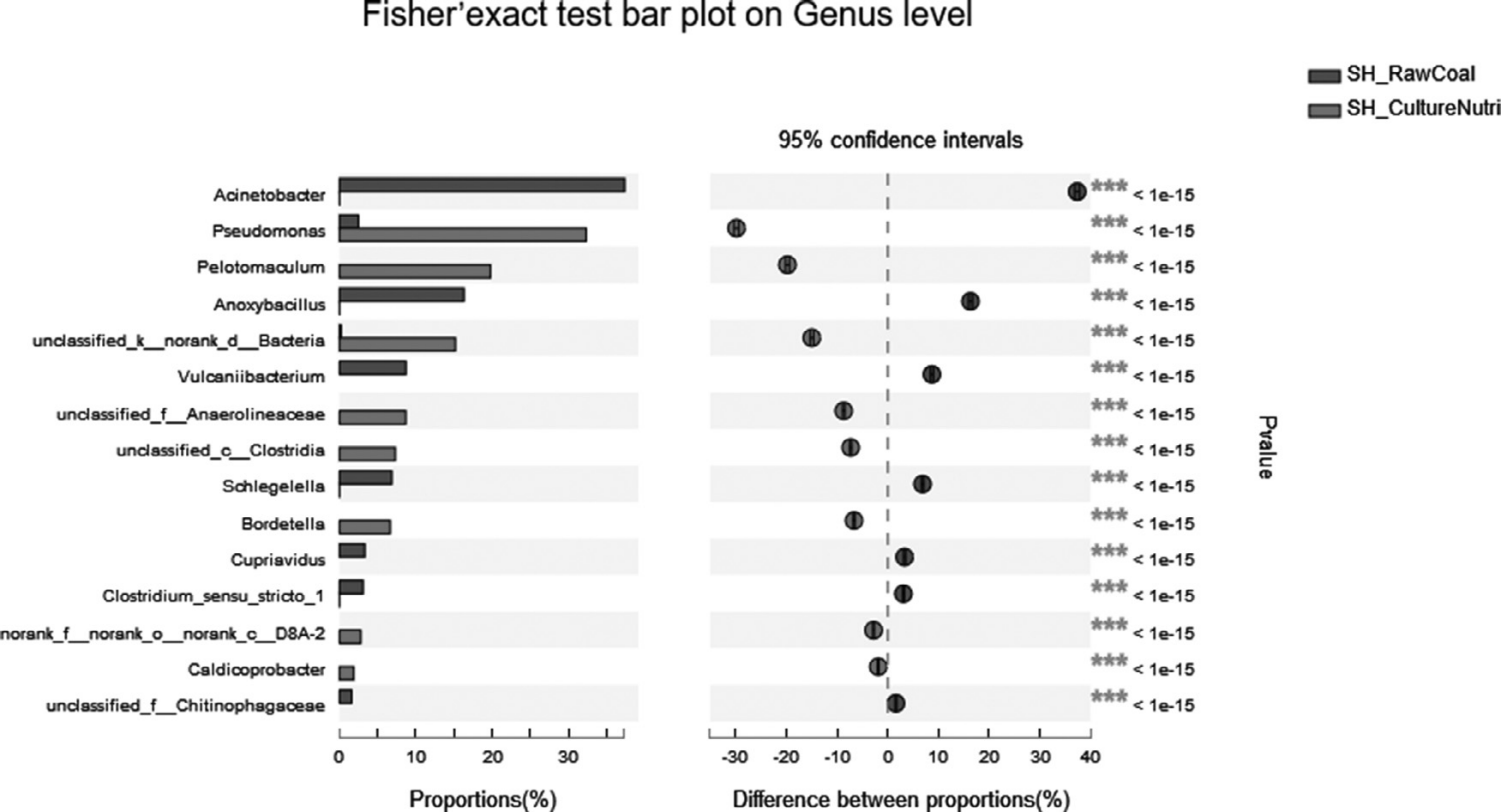
Continued.

The circular evolutionary tree selects the top 50 species in the total abundance of the species taxonomic level, uses ML (Maximum likelihood) to construct, and presents the phylogenetic relationship of the species in the form of a ring diagram. Software: IQ-TREE (version 1.6.8 http://www.iqtree.org/) (Fig. 5 B).

The Circos chart was drawn using the Genus taxonomy level, and the abundance of the samples in the group is calculated by summing up, and the relative abundance >0.01. Software: Circos-0.67-7 (http://circos.ca/) (Fig. 5 C).

Heatmap mapping was used the top 50 species of Species level, the second classification level: Phylum, and the species hierarchical clustering method: average. Software and algorithms: R language (version 3.3.1) vegan package (Fig. 5 D).

The comparative analysis of sample on Phylum and Genus level acquired the use of Fisher's exact test methodology to compare the abundance difference of species between the two samples, multiple test correction method: FDR, Cl calculation method: Diff Between Prop Asymptotic CC, confidence = 0.95. Software: stats package for R (version 3.3.1) and scipy package for python (Fig. 5 E, F).

## Ethics Statements

This paper comprises the research on the microbial degradation of coal and the stimulation of coalbed methane production. Likewise, the manuscript presents a dataset that consist of the author's original work and co-submitted with the manuscript “Primary studies on the effect of coal bio-gasification in situ in the Qinshui basin” (10.1007/s13202-021-01396-8) it is not currently being considered for publication elsewhere. The paper reflects the authors’ own research and analysis in a truthful and complete manner. In addition, the paper properly credits the meaningful contributions of co-authors and co-researchers. All sources used are adequately disclosed. All authors have been personally and actively involved in substantial work leading to the paper and will take public responsibility for its content.

## CRediT authorship contribution statement

**Dong Xiao:** Conceptualization, Methodology, Writing – original draft, Funding acquisition. **Mohamed Keita:** Investigation. **Cong Zhang:** Conceptualization, Data curation. **Enyuan Wang:** Methodology, Data curation. **Norberto Daniel Diaz:** Methodology, Writing – review & editing, Funding acquisition. **Junyong Wu:** Investigation. **Hailun He:** Data curation. **Jing Ma:** Investigation. **Essono Oyono Julien:** Investigation.

## Declaration of Competing Interest

The authors declare that they have no known competing financial interests or personal relationships that could have appeared to influence the work reported in this paper.

## Data Availability

Biodiversity Data of Sihe Coal Bio-gasification Experiment In-situ (Original data) (NCBI Sequence Read Archive).
